# DeepInsight-3D architecture for anti-cancer drug response prediction with deep-learning on multi-omics

**DOI:** 10.1038/s41598-023-29644-3

**Published:** 2023-02-11

**Authors:** Alok Sharma, Artem Lysenko, Keith A. Boroevich, Tatsuhiko Tsunoda

**Affiliations:** 1 Laboratory for Medical Science Mathematics, RIKEN Center for Integrative Medical Sciences, Yokohama, Japan; 2 Institute for Integrated and Intelligent Systems, Griffith University, Brisbane, Australia; 3 Laboratory for Medical Science Mathematics, Department of Biological Sciences, School of Science, The University of Tokyo, Tokyo, Japan; 4 Laboratory for Medical Science Mathematics, Department of Computational Biology and Medical Sciences, Graduate School of Frontier Sciences, The University of Tokyo, Tokyo, Japan

**Keywords:** Computational models, Data mining, Machine learning, Microarrays, Drug discovery

## Abstract

Modern oncology offers a wide range of treatments and therefore choosing the best option for particular patient is very important for optimal outcome. Multi-omics profiling in combination with AI-based predictive models have great potential for streamlining these treatment decisions. However, these encouraging developments continue to be hampered by very high dimensionality of the datasets in combination with insufficiently large numbers of annotated samples. Here we proposed a novel deep learning-based method to predict patient-specific anticancer drug response from three types of multi-omics data. The proposed DeepInsight-3D approach relies on structured data-to-image conversion that then allows use of convolutional neural networks, which are particularly robust to high dimensionality of the inputs while retaining capabilities to model highly complex relationships between variables. Of particular note, we demonstrate that in this formalism additional channels of an image can be effectively used to accommodate data from different omics layers while implicitly encoding the connection between them. DeepInsight-3D was able to outperform other state-of-the-art methods applied to this task. The proposed improvements can facilitate the development of better personalized treatment strategies for different cancers in the future.

## Introduction

Precision oncology is rapidly developing. However, only a very small percentage of patients can currently take advantage of it^1^. The risks of side effects can be reduced by improving the prediction rate of drug response from targeted therapy which would undoubtedly improve patients’ treatment. In this respect, drug prediction on in vivo datasets is a step towards clinical applicability. However, since in vivo data like The Cancer Genome Atlas (TCGA) repository (https://www.cancer.gov/tcga) has a scarcity of patient records and drug responses, it is difficult to train a model on in vivo datasets. Considering this challenge, Sharifi-Noghabi et al.^2^ trained their computational model on in vitro data and obtained predictability on in vivo data. In vitro projects have compiled datasets such as Genomics of Drug Sensitivity in Cancer (GDSC)^3^ and Cancer Cell Line Encyclopedia (CCLE)^4^. These datasets consist of multi-omics profiles such as gene expression, copy number alteration (CNA) and somatic mutations. Although gene expression datasets have shown to be very useful^3,5^, adding more omic layers could improve the predictability of pan-cancer models^2^. Nevertheless, the platforms of these datasets are different. Furthermore, there exists a scarcity of related samples in GDSC datasets. This augments the problem of adequately estimating a model on the training set of the data.

The application of modern machine learning (ML) methods to problems in biomarker discovery has allowed better prediction of response to treatment. Full scope of these advancements is too extensive to cover here, and the following recent review provides a more complete overview of proposed approaches^6^. To position this work among previous efforts, the field of anticancer drug response prediction can be viewed from the perspective of either the types of data used or the methodology applied. From the data-centric perspective, our method attempts to leverage patient multi-omics data, and as such, is closest to the following two approaches. In^2^, the MOLI method is proposed and learned on datasets from GDSC. It predicts response to a drug from TCGA and patient-derived xenograft (PDX)^7^ datasets. These collated datasets have multi-omics profiles: gene expression, CNA, and somatic mutation. The MOLI method reported the area under the curve (AUC) on seven datasets from TCGA and PDX. Their average AUC over the seven datasets was 0.63. Park et al.^8^ also constructed training and test dataset from GDSC and TCGA/PDX resulting in more than seven datasets and proposed the Super.FELT method. The performance of their method also included the seven datasets used in^2^, though some of the resulting test samples slightly differed due to methodology. Nonetheless, their average AUC over the seven datasets was reported to be 0.68. When we reimplemented the Super.FELT method on the MOLI test sets, an average AUC of 0.65 was obtained.

From the methodological angle, our approach is distinguished by the idea of applying convolutional neural networks to tabular data, more specifically by converting it to images. Some of the drug response prediction applications with similar methodology are described as follows. Omics data as images was used with convolutional neural networks (CNNs) in OmicsMapNet^9^. However, OmicsMapNet method was limited in that it relied on pathway maps for creating images. This means that the genes not mapped to particular pathways would be excluded. Another application of CNNs to drug response prediction was DeepIC50^10^, and this one-dimensional (1D) CNN method achieved the best result on the evaluated gastric cancer-specific dataset. As the idea of tabular image transformation gained popularity, more specialized methods for optimizing this transformation were developed, notably IGTD^11^, which was also applied to the task of drug response prediction. In contrast with these previous approaches that predominantly used a single type of data to create an image, this work specifically evaluates the potential of incorporating multiple, connected ‘omics layers within the same image through the use of multiple color channels to capture these relationships. Additionally, we illustrate how application of class-activation maps (CAMs) feature extraction can be used to facilitate interpretation of constructed CNN models. The theoretical basis of these proposed improvements is briefly explained below.

Typically, for tabular data, traditional ML methods are used for feature extraction, feature selection and classification problems. Essentially, a column vector of size $d\times 1$ is processed via ML methods for mining and extracting relevant information to perform classification or regression tasks. The complexity of the data in the medical industry is ever-increasing, stretching the boundaries of ML techniques for phenotype identification relevant to disease diagnosis and analysis. In this regard, choosing a small group of crucial genes or elements from a larger set has become a critical step. Furthermore, determining phenotypes via feature extraction and classification processes plays a vital role. Feature selection is crucial in many different types of research and is not just used in analyzing genomic data. Therefore, the procedures of feature selection, feature extraction, and classification have a major role in determining how reliable ML algorithms are at identifying a subset of genes with the correct phenotypes. However, traditional ML approaches ignore the neighbourhood information and presumptively consider each component of a sample to be mutually independent.

Two-dimensional CNNs, on the other hand, are a class of deep learning architectures that have demonstrated promising results and attracted considerable interest in all forms of image analysis. Deep learning techniques are well-known and applied in various research fields, including biological studies^12–19^. Contrary to traditional ML methods, CNNs undertake feature extraction and classification through their convolutional layers using an input image (a $p\times q$ feature matrix). CNNs are highly efficient, automatically extract features from spatially coherent pixels, requires fewer memory footprints enabling deeper network with fewer parameters because of sharing of weights across nodes^20^, detect higher-order statistics and non-linear correlations, and use less samples to provide promising performance. An image in a local region is made up of spatially coherent pixels, which means that the nearby pixels share similar information. This neighbourhood information is extracted from the adjacent pixels by the layers of CNNs. Therefore, the adjacent pixels of a 2D input feature matrix should have adequate coherence in order to achieve meaningful output from a CNN.

Unlike other ML approaches, the order of nearby pixels in an image used by CNN is no longer independent. In order to arrange tabular data, e.g. omics data, to an image, DeepInsight^21^ pioneered through the element arrangement step and followed by mappings. Thereafter, automatic feature extraction and classification processes via CNNs are conducted. In the 2D pixel frame, the elements or genes are located according to how similar they are, and then the values are mapped to these locations to create the desired layout. With this method, any non-image sample can be converted into a set of images that can be used by CNNs. According to our understanding, it was the first method for transforming different types of non-image input into picture forms for the use of CNN architecture. This method of transforming tabular data to images has been applied and/or further developed in various fields^22–28^. DeepInsight was a component in the Kaggle.com competition hosted by MIT and Harvard University that secured rank 1 on the leaderboard^29^.

**Figure 1:**
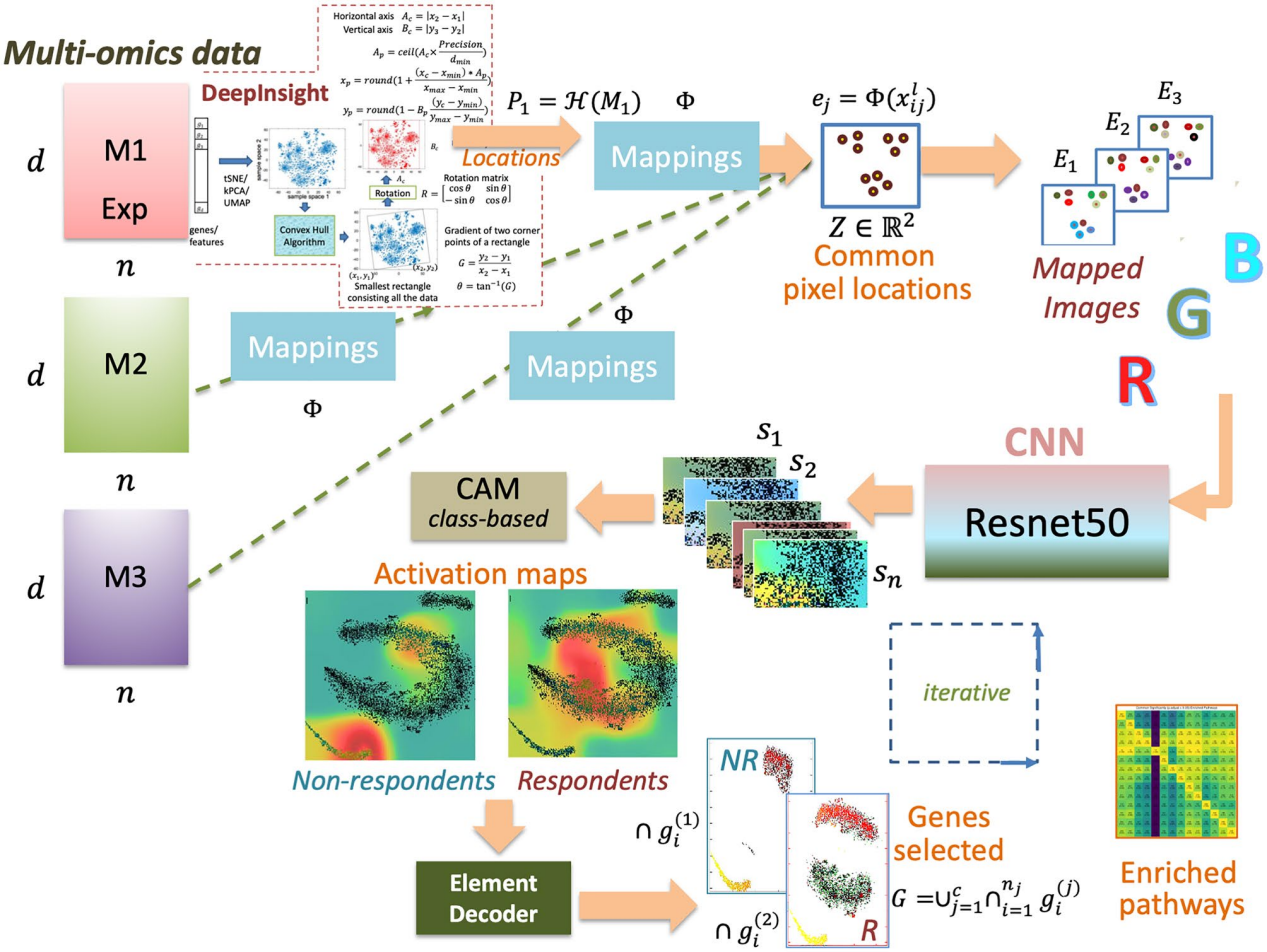
An overview of the DeepInsight-3D model. From the left multi-omics layers are processed via DeepInsight methodology and common pixel locations are found. After mapping omics data, corresponding images are constructed, which are processed to a convolutional neural network. Afterwards, CAM is used to find activation regions and element decoder is used to find a subset of genes.

The proposed method is based on the DeepInsight method^21^ and the DeepFeature method^30^. The DeepInsight-3D model considers the limitations of DeepInsight and DeepFeature and further extends their utility for multi-layered data. The overview of the proposed model is given in Fig. 1 (see Methods for the details). DeepInsight can convert a feature vector of size $d\times 1$ to an image of size $p\times q$ following its pipeline. It can effectively deal with data of one kind. However, multi-omics data has several layers and, in this case, DeepInsight is restrictive to performing the transformation. We looked into this limitation and expanded the algorithm for multi-layers. Therefore, a sample ${s}_{1}$ having $L$ layers of column vectors ${c}_{1}$, ${c}_{2}$ … ${c}_{L}$ of size $d\times 1$ each, can now be transformed to a tensor-like shape of size $p\times q\times L$ using DeepInsight-3D. This enables the element arrangement and feature mapping for multi-layered cases. If the number of layers is restricted to three, then it will give a colored image of a sample ${s}_{1}$. Using dimensionality reduction techniques, such as t-SNE^31^, DeepInsight-3D arranges similar elements from multi-layered data together in a 2D pixel frame and then performs element mappings of all the three layers. DeepInsight-3D can transform in two ways: (1) transformation using the dominant layer, where the most informative layer dictates the pixel locations and the values of other layers are mapped to these pixel locations. (2) The transformation by the equal contribution of layers. This approach finds the pixel locations in two stages; i.e., the element arrangement step is performed two times. In this way, the proposed DeepInsight-3D model, first converts multi-omic or multi-layered data into corresponding images that are organized and colored, and then applies a convolutional neural network (CNN) with automatic feature extraction ability. Moreover, the element or feature selection of the transformed data ($p\times q\times L$) is carried out via the class-activation maps^32^ and the Element Decoder step. The feature selection is achieved by decoding activations obtained by the last ReLu layer of the CNN architecture.

Considering a multi-omics dataset $M$ having $n$ samples, $d$ dimensions (number of genes or elements) and 3 layers, where $d\gg n$. This leads to a small sample size problem. However, since DeepInsight-3D converts omics vectors to colored images using transformation $\mathcal{H}$ which handles groups of genes as features via element arrangement and direct mapping of genes to pixel locations, making the model robust compared to the limited sample size. For CNN the weights are not independent and it can bypass the curse of dimensionality problem^33^. While the sample sizes of datasets are minimal, DeepInsight-3D with CNN can still perform well if the model is appropriately tuned.

Anticancer drug response is highly complex because not only tissue of origin leads to high diversity of subtypes, but also an ever-evolving landscape of somatic mutations make each cancer essentially unique. Combinations of mutations give rise to different strengths and weaknesses and therefore modern treatments commonly rely on biomarkers to identify which drugs are most likely to be effective^34^. Single-gene biomarkers are now widely and successfully used, most notably BRCA1/BRCA2, ER and PR gene mutations for breast cancers^35^, BRAF and KRAS mutations in colorectal and lung cancers^36^ and PD-1/PD-L1 expression for targeting immunotherapy^37^. However, biological processes often arise through complex interactions of different genes, cell types and signaling molecules that are far more challenging to profile, but vital for making further improvements to treatment^38^. Based on these previous results it is now clear that modern machine learning approaches can indeed deliver better predictive performance, but as a trade-off, recovering biological mechanisms that underpin such models still remains challenging. More advanced methods, like deep learning, can automatically perform feature extraction and engineering to deal with high dimensionality of the input data as well as discover interactions, and the potential of such methods for complex biomarker discovery is now well-recognized^39^. Interpretation of these discovered relationships is important both for increasing confidence of doctors in using such tools in the clinic and for identifying research leads for future improvements. In this work we demonstrate that some of this complexity can be interpreted by mapping feature importance extracted by commonly-used methods like CAM to the biological pathways. Importantly, unlike in classical pathway enrichment analysis, the genes are extracted based on the magnitude of their contribution to the “answer” of the model rather than simply the level of expression. Our analysis shows that the model does indeed extract groups of genes that are non-random and have meaningful correspondence to different types of pathways, some of which were previously linked to anti-cancer drug response mechanisms.

**Figure 2:**
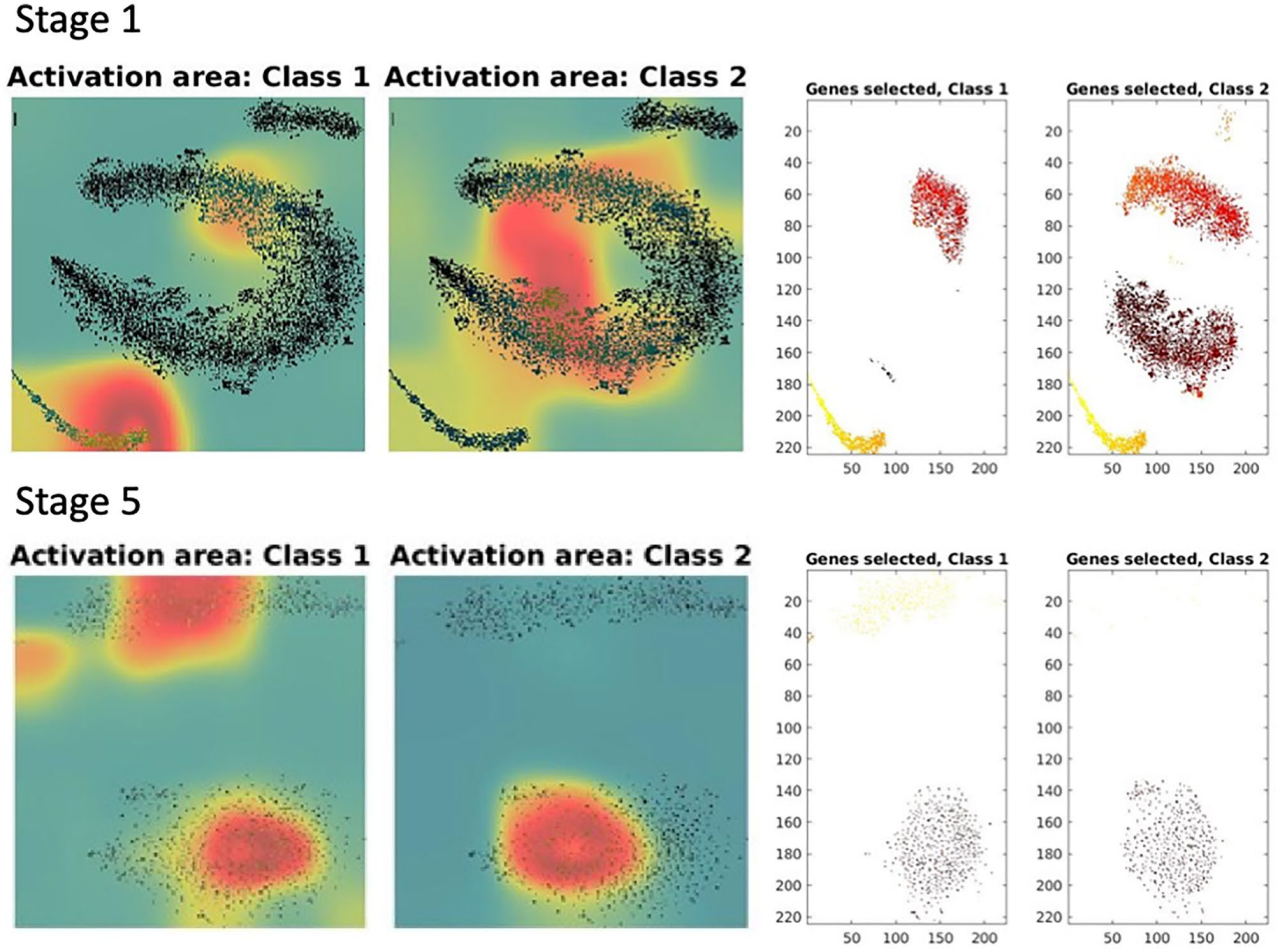
Activations and gene selection on Paclitaxel at Stage 1 and Stage 5. Class 1 is non-respondents and Class 2 is respondents. The left side of the figure depicts the activations in Class 1 and Class 2, and the right side illustrates the selected genes in the respective classes.

The contributions of this work are as follows. DeepInsight-3D pipeline is presented where classification and feature selection can be performed for multi-layered non-image samples (or tabular data) through the application of CNNs. Two ways of image construction are introduced, (1) by mapping elements to the pixel locations of the dominant layer (shown in Fig. 1), and (2) by mapping elements to the pixel locations obtained by giving equal importance to all the three layers (implemented in the DeepInsight-3D package as an option). Element decoder is implemented to find genes or elements from the activation maps. We also demonstrate how the developed system can be used to interpret the CNN model and report on the identified key genes and biological processes identified as important for drug response prediction by respective models.

## Results

### Performance evaluation

The datasets and empirical organization have been summarized here. The details are given in the Experimental Setup section and Supplement Files. Seven datasets from the GDSC resource are used for the training/validation of the model. The number of total training samples for Paclitaxel is 389, Gemcitabine is 844, Cetuximab is 856, Erlotinib is 362, Docetaxel is 829 and Cisplatin is 829. The training set was divided into 90:10 ratio for separate smaller training set and validation set. The independent test sets are taken from PDX/TCGA resource. The number of test samples for Paclitaxel (PDX) is 43, Gemcitabine (PDX) is 25, Cetuximab (PDX) is 60, Erlotinib (PDX) is 21, Docetaxel (TCGA) is 16, Cisplatin (TCGA) is 66 and Gemcitabine (TCGA) is 57. Multi-omics data with expression, CNA and mutation layers are transformed into colored images by Deepinsight-3D. In all the datasets, the number of genes was over 13,000, which was much larger than the number of samples (less than 900). This leads to the high-dimensionality problem. However, since for CNN the weights are not independent, it is not affected by the curse of dimensionality^33^. Information loss occurs when two or more than two pixels coincide in the same position of the image framework, compelling two or more than two gene values to attain an average in this particular pixel location.

Next, since expression data contains the majority of information, it has been used to find the pixel locations (Fig. 1). All the layers are utilized to map their values on these common pixel locations. DeepInsight-3D offers to use of manifold techniques, such as t-SNE, UMAP, kernel-PCA and PCA. In our experiments, we found t-SNE showing better results than other techniques, which is why t-SNE was selected. The technique, t-SNE, is not applied in the usual manner, as the visualization of samples in the 2D plane is not required. We want to find genes in the 2D plane, so transposition process is performed prior to applying t-SNE. This would provide points in the Cartesian coordinates corresponding to the genes or elements. After that, the convex-hull algorithm is used to find the minimum square around the plotted points (see Fig. 1). However, the square and the points within, need to be rotated to align with the horizontal and vertical axes. For this reason, a rotation matrix has been used for alignment. Afterwards, the rotated Cartesian coordinates framework is constructed into pixel coordinates (with fixed rows and columns). This process would give pixel locations from the expression layer. Then the expression data will be mapped to these locations. These steps are not followed for the other two layers (CNA and mutation). However, following the previous steps, their values will be mapped on the acquired pixel location. Once the mapping of all the three layers is completed, a colored image corresponding to a sample of multi-omics data can be visualized. In this work, a pre-trained ResNet-50 has been used to train the transformed images from the seven multi-omics datasets. The hyperparameters were adopted from DeepInsight version 2 (please see the Methods section and Supplement File 1 for more details). A Bayesian optimization technique was also applied for a drug dataset (Cisplatin) to improve the classification performance.

Two recently developed methods, MOLI and Super.FELT, were used as benchmark methods. Other than these methods, we have also compared with non-negative matrix factorization (NMF), feed-forward net, and Geeleher et al.^40^ as reported in^2^. Moreover, a comparison was made with autoencoder (AE), artificial neural network after feature selection (ANNF), AutoBoruta Random Forest (AutoBorutaRF)^41^ and SVM^42^ as reported in ^8^. All these methods were compared with many preceding algorithms and showed superior performance. The test set configurations (in terms of the number of samples) were kept the same for a fairer comparison. However, the test samples may slightly differ for the methods used in the Super.FELT work. The AUCs were computed for all possible drugs-method combinations and are given in Table 1.

**Table 1:** A comparison of DeepInsight-3D for drug response prediction with multi-omics profiles using test AUCs.

Drug	Paclitaxel	Gemcitabine (PDX)	Cetuximab	Erlotinib	Docetaxel	Cisplatin	Gemcitabine (TCGA)	Average
Early inetgration via NMF	0.24	0.56	0.53	0.28	0.39	0.40	0.58	0.43
Feed forward net	0.68	0.48	0.43	0.37	0.69	0.44	**0.65**	0.53
Geeleher	0.52	0.59	0.58	0.67	0.59	0.62	0.53	0.59
AE*	0.44	0.47	0.42	0.33	0.50	0.46	0.50	0.45
ANNF*	0.64	0.69	0.43	0.65	0.64	0.68	0.57	0.61
AutoBorutaRF*	0.46	0.45	0.17	0.17	0.42	0.45	0.53	0.38
SVM*	0.49	0.64	0.41	0.67	0.53	0.47	0.47	0.53
MOLI	**0.74**	0.64	0.53	0.63	0.58	0.66	**0.65**	0.63
Super.FELT	0.64	0.65	0.55	0.76	0.64	**0.73**	0.61	0.65
DeepInsight-3D	**0.74**	**0.72**	**0.71**	**0.85**	**0.78**	0.68	0.53	**0.72**

The test samples are the same for all the comparators. The highest results are marked with bold fonts.

*These methods are executed on a slightly different test sets (but similar enough for comparison) as reported in the Super.FELT paper.

It can be observed from Table 1 that for Paclitaxel, MOLI and DeepInsight-3D produced promising AUCs. For Cisplatin, Super.FELT had the highest, and for Gemcitabine (TCGA), MOLI produced the highest. For the remaining 4 drugs, Gemcitabine (PDX), Cetuximab, Erlotinib and Docetaxel, DeepInsight-3D produced the highest AUCs. The average AUC over all the seven datasets for the MOLI method was 0.63 and for Super.FELT was 0.65. DeepInsight-3D produced an encouraging average AUC of 0.72. For other baseline methods, the average AUC reported are 0.43 for NMF, 0.53 for the feed-forward net, 0.59 for Geeleher, 0.45 for AE, 0.61 for ANNF, 0.38 for AutoBorutaRF and 0.53 for SVM. The confusion matrix of the DeepInsight-3D results over the seven datasets can be seen in Table S3 (Supplement File 1). In brief, the true positive rate (TPR) for Paclitaxel (PDX) was 24/38, Gemcitabine (PDX) was 10/18, Cetuximab (PDX) was 28/55, Erlotinib (PDX) was 13/18, Docetaxel (TCGA) was 6/8, Cisplatin was 1/6 and Gemcitabine was 10/36. The true negative rate (TNR) in the same order was 3/5, 5/7, 5/5, 2/3, 4/8, 46/60 and 18/21. The additional evaluation parameters such as average accuracy, sensitivity, specificity, and F1-score over all the drug datasets was 0.62, 0.52, 0.73 and 0.58, respectively, by the DeepInsight-3D method (please refer to Table S7, Supplement File 1 for details). For uncertainty analysis, repeating the same configuration would not significantly affect the results and the results remain same as reported in Table 1. Nonetheless, the AUCs over three repetitions for drugs Paclitaxel (PDX), Gemcitabine (PDX), Cetuximab (PDX), Erlotinib (PDX), Docetaxel (TCGA) and Gemcitabine (TCGA) were 0.74, 0.72, 0.71, 0.86, 0.78, and 0.53. For Cisplatin (TCGA), Bayesian optimization technique with 10 objective functions were used and the best, for which the validation error was minimum, was selected. However, when we change the configuration of DeepInsight-3D method as an ablation study, we observe the variation in the results, which are reported in the summarized way in Figure S6 (Supplement File 1) and in details in Supplement File 3.

### Feature selection to identify genes of interest

DeepInsight-3D can also perform feature selection via class-activation maps (CAMs) to identify genes of interest for each dataset. Since the data dimensionality is very large compared to the number of samples available, there is a high chance of producing an unstable model estimate. Furthermore, not all genes can be well represented in a limited pixel-framework. Appropriate feature selection would reveal background scientific mechanisms. Therefore, we applied an iterative way of conducting feature selection. Gene selection can be performed in 3 ways, (1) considering CAM values for every training sample, (2) taking an average of CAM over training samples, and (3) class-based CAM (described in the Methods section) where the average over a particular class is considered. In this work, class-based CAM has been applied for gene selection. Table S5 (Supplement File 1) depicts the number of genes selected for each drug dataset (non-respondents and respondents). For parameters related to feature selection, see Table S4 (Supplement File 1) and feature selection procedure in Figure S1 (Supplement File 1). The activation maps are shown in Fig. 2. Here the activations of Paclitaxel dataset at stage-1 and stage-5 (last stage) are illustrated. Only the training set of Paclitaxel has been used to find the activations. The selected genes are also depicted on the right-hand side of Fig. 2. The activations for all the seven drugs are depicted in Figure S2 (Supplement File 1).

### Pathway-centric context of discovered gene sets

Gene sets identified as important by each drug-specific model were mapped to Reactome^43^ pathways and QIAGEN Ingenuity Pathway Analysis (IPA) knowledgebase^44^, as described in the methods section. This analysis has revealed that there were both a unique as well as a shared component that was recovered as important for all drugs. The overlap between drug-specific gene sets (Figure S3, Supplement File 1) was very low and consequently we have found that there was very low coverage of relevant common pathways in Reactome database, although this analysis did identify a small number of significantly enriched pathways for most of the drugs. Therefore, we have done additional analysis using IPA software, which is backed by a much larger database and has more pathway definitions with higher levels of granularity. In several cases most significantly enriched subsets have previously been reported in literature as being linked to particular drugs or that class of drugs. This suggests that the proposed system does have some functionality to not only improve quality of drug response prediction, but also allow discovery of meaningful biological processes that may be involved. Summary results are available as a Supplement File 2 (Tables S8–S12); and relevant key findings are summarized below.

Overall, seven evaluations were performed across six different drugs and two independent datasets (TCGA and PDX). One drug, gemcitabine, was profiled in both of these datasets. As described in detail in the methods section, our model interpretation approach allows recovery of class-specific important feature sets, therefore in total fourteen sets were generated and analyzed—i.e., separate gene sets were produced for responders and non-responders for each drug/data source combination. Note that for the purposes of interpretation, feature sets identified for the responder and non-responder classes are of equal interest and relevant gene combinations may be allocated to either or even both of the sets. Profiled drugs have covered a range of commonly exploited mechanisms of action: growth factor blockers, microtubule-binding and different forms of DNA damage.

The initial analysis that was based on the Reactome pathway definitions did not identify substantial commonalities between significantly enriched pathways across all of the drugs (Figure S4, Supplement File 1), and furthermore very few results were returned for some of the gene sets. This is likely caused by the relatively small size of the dataset and, in particular, very small number of responders. Although DeepInsight approach does allow for better model generalization in smaller datasets, some effect of noise is still unavoidable in such cases and can lead to greater variability of feature selection from a pool of all potentially usable options. As Reactome analysis did indicate the presence of some significant pathways, a follow-up analysis was done in IPA to explore whether the relatively low number of significant hits was solely due to the challenging dataset or relatively sparser coverage offered by the database used in the evaluation.

**Figure 3:**
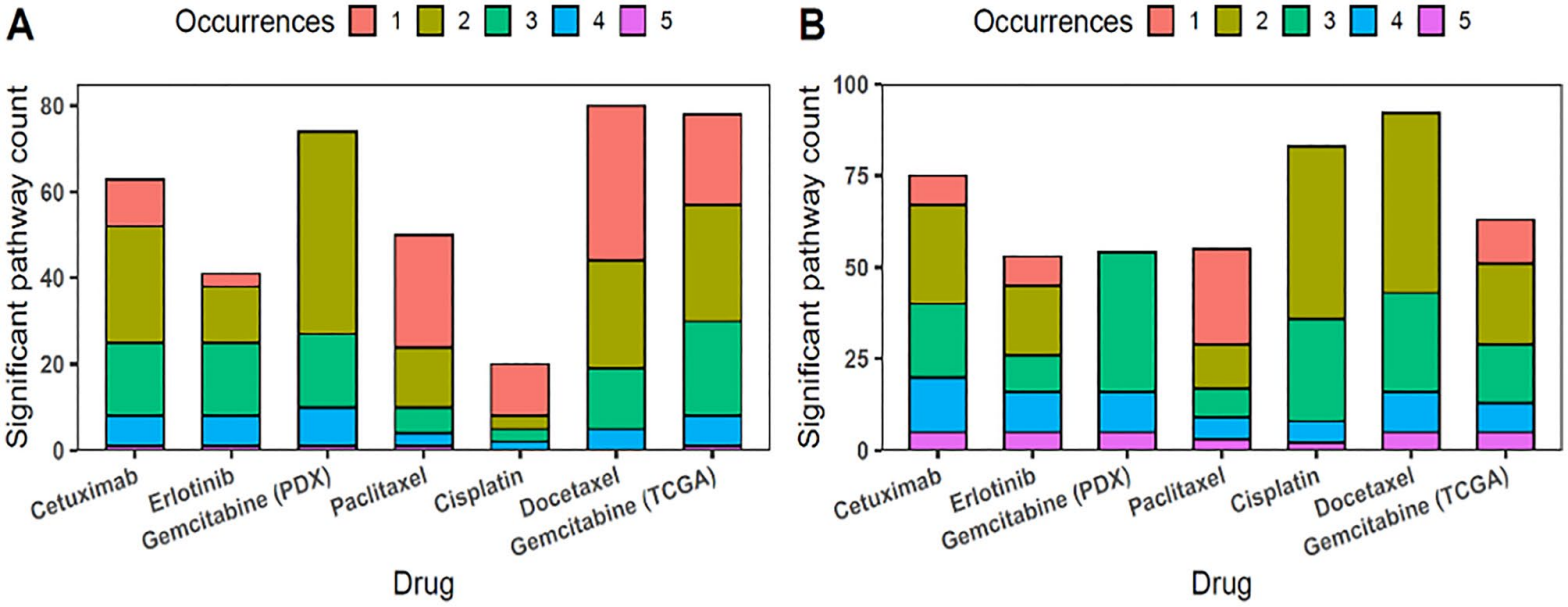
Annotation of selected genes to IPA pathways across all drug-specific classifiers for responder (**A**) and non-responder (**B**) classes. Occurrences bars show how many times that part of annotations is also found in sets for other classifiers. Data were analyzed through the use of QIAGEN Ingenuity Pathway Analysis (IPA), 2022 Summer Release (July) data, with client 01–20-04 (installed on 14 Sep 2021).

**Figure 4:**
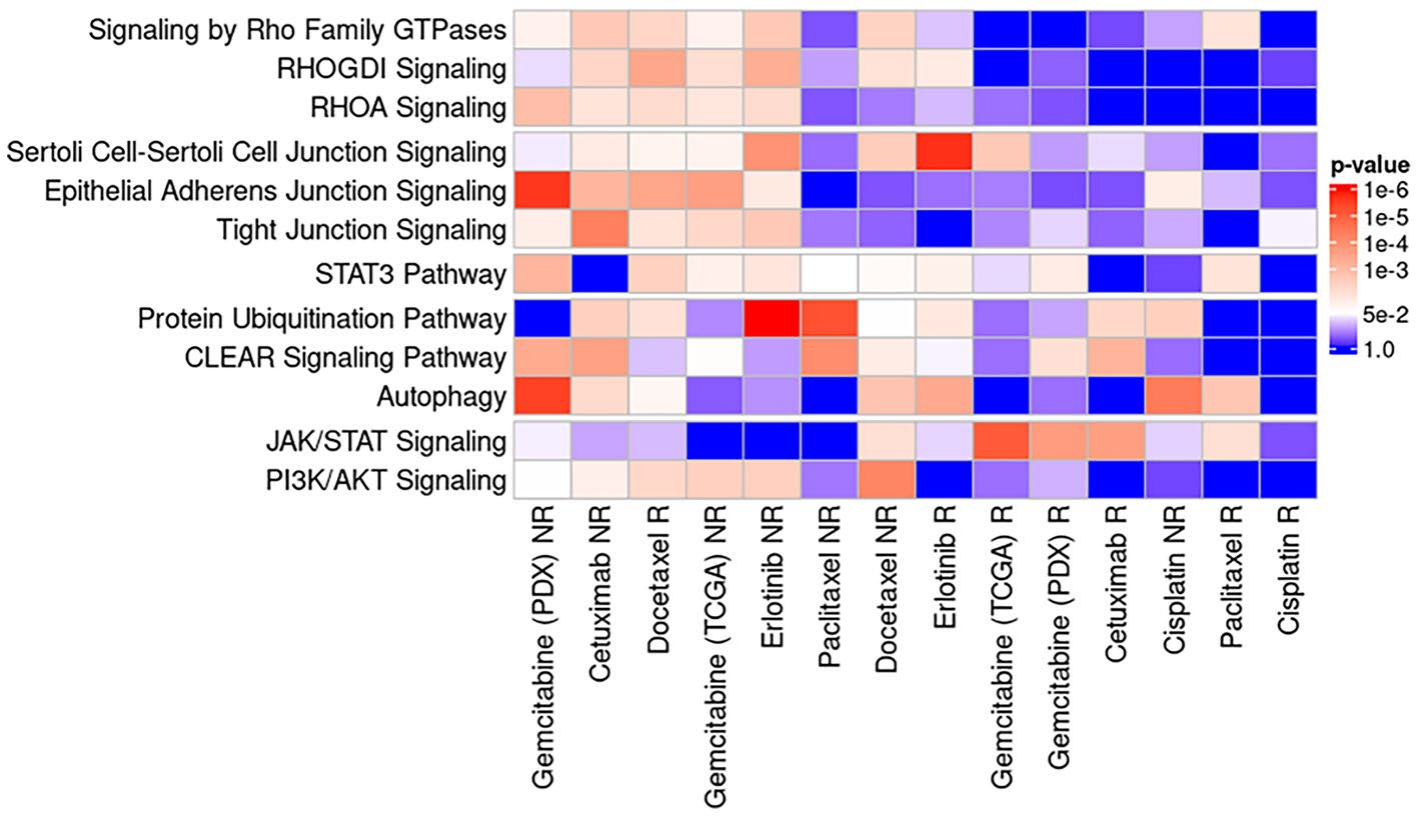
Notable pathways identified as significant by metabolic pathway enrichment using IPA software in gene sets of importance in multiple prediction models. Data were analyzed through the use of QIAGEN Ingenuity Pathway Analysis (IPA), 2022 Summer Release (July) data, with client 01–20-04 (installed on 14 Sep 2021).

Metabolic pathway enrichment analysis using IPA software has identified 269 (responders) and 342 (non-responders) non-redundant canonical pathways that were significant in at least one of the drug-specific sets. None of the pathways were found to be significantly enriched in all 7 of the drug-specific sets in either responder or non-responder categories (Fig. 3). However, a number of key common pathways were recovered for 5–4 sets, suggesting that those may be potentially important mechanisms of multidrug resistance (Fig. 4). Full results of this analysis, including log-transformed significance values for individual pathways are made available in Supplement File 2 (Tables S8–S12). One of the standout features of these results is functional consistency of the results across multiple classifiers. Given the size of the datasets, functions independently identified as important multiple are least likely to be due to overfitting or noise, therefore, we have decided to dissect these functional categories further and check if they have been previously reported to be linked to multidrug resistance. Some notable examples include “STAT3 Pathway” that was significantly enriched among both responder (Docetaxel *p* = 0.004; Gemcitabine (PDX) *p* = 0.017; Erlotinib *p* = 0.022; Paclitaxel *p* = 0.01) and non-responder (Gemcitabine (TCGA) *p* = 0.02; Docetaxel *p* = 0.04; Gemcitabine (PDX) *p* = 0.0008; Erlotinib *p* = 0.011; Paclitaxel p = 0.049) gene sets. STAT3 gene is often highly expressed in various cancers and can lead to therapy resistance by upregulating expression of anti-apoptotic proteins, stimulating DNA repair and cell proliferation^45^. Functionally closely related pathways “PI3K/AKT Signaling” (responders: Docetaxel *p* = 0.004; Gemcitabine(PDX) *p* = 0.017; Erlotinib *p* = 0.022; Paclitaxel *p* = 0.011; non-responders: Gemcitabine(TCGA) *p* = 0.004; Docetaxel *p* = 6.5 × 10^–5^; Gemcitabine(PDX) *p* = 0.048; Erlotinib *p* = 0.004; Cetuximab *p* = 0.021) and “JAK/STAT Signaling” (responders: Gemcitabine(TCGA) *p* = 8.6 × 10^–6^; Gemcitabine(PDX) *p* = 2 × 10^–4^; Cetuximab *p* = 2.3 × 10^–4^; Paclitaxel *p* = 0.008; non-responders: Docetaxel *p* = 0.008) were also found important in multiple sets and are likewise well-known to be involved in multidrug resistance^46,47^. All of these pathways are well-known to play a key role in cancer cellular growth, survival, and resistance to treatments. Likewise, three variants of Rho GTPase family signaling pathways were recovered multiple times among the non-responder gene sets, which are an important mechanism for the upstream activation of JAK/STAT pathway^48^ and cancer progression^49^. These results are in line with current understanding of the pivotal role these signaling pathways play in various drug resistance mechanisms^50^.

Another common theme was the potential importance of protein degradation and recycling mechanisms for cancer drug resistance. Functionally-linked ubiquitination (responders: Docetaxel *p* = 0.009; Erlotinib *p* = 0.014; Cetuximab *p* = 0.005; non-responders: Cisplatin *p* = 0.003; Erlotinib *p* = 4.8 × 10^–7^; Cetuximab *p* = 0.004; Paclitaxel *p* = 5.7 × 10^–6^), autophagy (responders: Docetaxel *p* = 0.031; Erlotinib *p* = 4.1 × 10^–4^; Paclitaxel *p* = 0.002; non-responders: Docetaxel *p* = 0.002; Cisplatin *p* = 3.7 × 10^–5^; Gemcitabine (PDX) *p* = 3.4 × 10^–6^; Cetuximab *p* = 0.006) and lysosome regulation pathways “CLEAR Signaling Pathway” (responders: Gemcitabine (PDX) *p* = 0.009; Cetuximab *p* = 7.2 × 10^–4^; non-responders: Gemcitabine (TCGA) *p* = 0.044; Docetaxel *p* = 0.017; Gemcitabine (PDX) *p* = 4.4 × 10^–4^; Cetuximab *p* = 2.6 × 10^–4^; Paclitaxel *p* = 9.3 × 10^–5^) were found multiple times among the non-responder gene sets. Ubiquitination marks proteins for proteolysis and this mechanism is usually preserved largely intact in cancer cells, suggesting functional essentiality^51^. Both lysosome^52^ and ubiquitination-meditated proteasome^53^ are believed to be involved in multidrug resistance. These processes are part of a broader set of autophagy processes that in turn lead to cellular rejuvenation, stress resistance and medication of anti-drug responses on a cellular level^54^.

The last commonly represented theme was the presence of several pathways to do with extracellular structures and cell adhesion. Some specific examples in this category included “Epithelial Adherens Junction Signaling” (responders: Gemcitabine (PDX) p = 0.028; non-responders: Gemcitabine (TCGA) *p* = 2.4 × 10^–4^; Cisplatin p = 0.019; Gemcitabine (PDX) *p* = 2.5 × 10^–6^; Erlotinib *p* = 0.015; Cetuximab *p* = 8.1 × 10^–4^), “Sertoli Cell-Sertoli Cell Junction Signaling” (responders: Gemcitabine (TCGA) *p* = 0.002; Docetaxel p = 0.026; Erlotinib *p* = 2 × 10^–6^; non-responders: Gemcitabine (TCGA) *p* = 0.026; Docetaxel *p* = 0.003; Erlotinib *p* = 1.2 × 10^–4^; Cetuximab *p* = 0.016) and “Tight Junction Signaling” (responders: Docetaxel *p* = 0.010;non-responders: Gemcitabine (TCGA) *p* = 0.005; Gemcitabine (PDX) *p* = 0.020; Erlotinib *p* = 0.002; Cetuximab *p* = 4.7 × 10^–5^). Interestingly, multiple pathways in this broader functional category were identified in both Reactome and IPA-based enrichment analyses. These processes are important for formation of tumor microenvironment, interactions with the immune system as well as cancer cell dissimilation/metastasis. Some of the identified pathways were previously reported to be involved in drug resistance, for example paxillin was reported to be involved in tyrosine kinase inhibition in lung cancer^55^ and was significantly enriched for both of the tyrosine inhibitor drugs sets (Cetuximab and Erlotinib) in our analysis. More generally, tight junctions play a role in drug efflux^56^ and are important for anoikis and metastasis^57^. Extracellular matrix can confer a cell adhesion-mediated drug resistance in some cancers, like glioblastoma^58^ and small cell lung cancer^59^. However, the potential broader roles in anti-cancer drug resistance are currently not very well defined and these results hinting at prominent involvement highlight a need for further study.

Among the pathways found to be important and significant across several drugs two appear to be relatively unexplored with respect to selected drugs. The first example is “Tryptophan Degradation X”, which was significant for Gemcitabine (TCGA), Erlotinib (PDX) and Cetuximab (PDX). This result is of interest because tryptophan metabolism was proposed as a source of potential targets for future anti-cancer drugs^60,61^, and better understating of its interplay with established treatments may facilitate these developments. Another pathway of interest was “Clathrin-mediated Endocytosis Signaling”, which was significant for the same set of drugs. Endocytosis is a process by which molecules on the surface of a cell, like nutrients and signaling molecules, are internalized, mostly for subsequent post-processing or degradation. As a number of cancer driver and drug target proteins are located on the cell surface, it plays an important but very complex role in cancer^62^, and clathrin is one of the key components of this process^63^. Likewise, the role of this pathway in mediating response to these particular drugs still appears to be unclear. Of course, more detailed follow up of these links will be necessary to verify the observed effects and understand their roles further.

## Discussion

Response to cancer treatment is highly complex as it is underlain by both genomic and somatic variations^64^, which can interact to give rise to yet more variation across all other ‘omics. This inevitably leads to very high degree of tumor heterogeneity across patients due to their genomic characteristics^65^, which in turn leads to often vast differences in efficacy of specific treatments. Better understanding of these factors is essential for improvements in drug response prediction that will not only allow more patients to benefit through better targeting of available therapies^1^ but also avoid potential adverse effects^66,67^. In order to manage the complexity, increase scale and minimize risks to patients, drug response is often studied on large in vitro datasets^4^. The discovered mechanisms are then taken forward to pre-clinical models and eventually to clinical trials. One of the key aspects, therefore, is translatability of these results to actual better outcomes in cancer patients^68^. Specifically in the case of computational predictive models, the performance achieved on the in vitro training data must be comparable to that on the real patient data. Importantly, although in vitro high-throughput screening is inevitably required, the number of possible genomic factors and drug combinations make it impossible to exhaustively test all possible options^69^. Therefore, statistical and machine learning models are increasingly necessary to mine these huge datasets and discover novel clinically usable patterns^70^. In turn, these types of analyses are also used to streamline experimental work and therefore reduce the amounts of data that needs to be collected. As a possible additional benefit, more advanced machine learning methods can help to identify combinations of factors that contributed to making a particular prediction in each case and in that way facilitate the identification of pharmacological functions of these targets^71^.

In this context, DeepInsight-3D offers two important capabilities. First one is an ability to make accurate predictions of drug response from the high-dimensional ‘omics data. As the cost of collecting such samples continues to decrease, this information is now increasingly used by doctors to make better treatment decisions, creating a need for relevant tools. Potentially, ‘omic-based predictions can be combined with other clinical data as part of the decision process. The second capability is the discovery of underlying features of importance that may then facilitate identification of relevant mechanisms. Deep learning methods have an inherent ability to discover non-linear and interacting features when sufficient data is available—and therefore can expand the range of characterizable features beyond what is possible using less complex methods. DeepInsight-3D extends the versatility of applying CNN to multi-layered tabular data. In this work, DeepInsight-3D provided very encouraging results on drug response multi-omics data. DeepInsight-3D was able to produce an average AUC of 0.72 over seven drug response datasets which is encouraging compared to competing methods in the literature.

The promising performance of DeepInsight-3D could be attributed to the following: (1) The inclusion of neighborhood information through the placement of similar elements together and dissimilar ones apart. (2) Three different layers can be combined into colored images, keeping all the possible information as kernels. (3) The interconnectivity of deeper networks (CNNs) taps into the non-linear connectivity between elements which shallow or traditional machine learning methods could miss. However, at the same time as one of limitations, a limited number of samples affects the confidence of the model, which could lead to unsatisfactory performance. Therefore, it is generally opted to have ample training samples for better estimation of the model.

Deep learning nets, such as CNN, have many merits, such as automatic feature extraction, finding hidden structures from hyper-dimensional data, finding higher-order statistics of image and non-linear correlations, economical use of neurons for large input sizes allowing much deeper networks are plausible with fewer parameters^20^, and a parsimonious memory footprint. These properties of CNN can be integrated with the inception of DeepInsight-3D for non-image tabular data with multi layers.

In machine learning techniques for tabular data, any two features are considered mutually independent. However, DeepInsight tries to establish a relationship through the element arrangement step by positioning similar elements together and dissimilar ones apart^21^. DeepInsight-3D further extends this property to multi-layered data. Moreover, the application of DeepFeature is extended. DeepFeature enables a powerful means for the identification of biologically relevant gene sets and provides methodological basement for "explainable AI"^30^. This has been integrated with DeepInsight-3D to simultaneously identify elements for multi-layered data.

Functional annotation of gene sets found to be important for particular classifications was largely in line with pathways and mechanisms previously reported in the literature. Notably, despite very small training sets, there was remarkable degree of consistency across discovered functional categories among different classifiers. These functions also corresponded to well-known multidrug resistance mechanisms as well as core cancer pathways that are linked to general “fitness” of cancerous cells. Since drug response is highly complex and determinants are highly drug-specific these processes would not be expected to be fully predictive of drug response on their own. However, an ability to discover this relevant subset of functions across multiple datasets suggests that DeepInsight transformation may prove particularly useful in comparative analysis across multi-omics datasets and lead to discovery of common mechanisms. Another notable pattern in these results was that relatively few functions were completely unique to particular drugs. This is likely due to the way relatively small sample size of the datasets interacts with convolution done when data is presented as image, which would favor more consistently present features in such cases.

In this work, DeepInsight-3D is used for multi-omics datasets. However, the proposed method is not limited to omics data. It can handle different kinds of multi-layered tabular data (as long as the elements and samples of diverse layers are arranged in the same order). This method does not require any specific biological information such as chromosome locations and visualizes non-image data through multi-layered mappings.

Although the results were promising, the severe scarcity of training and test samples hindered getting a reasonable model estimate. The same was true for MOLI and Super.FELT methods, as their results were sensitive to parameter tuning. In general, CNN works very well when the samples are sufficiently large. However, this was not the case in the work. Nonetheless, all these methods provided a good platform in this direction. DeepInsight-3D can perform sufficiently well when the sample size is sufficient such as in the case of single-cell analysis. This would be our future direction of work.

## Conclusions

The proposed method, DeepInsight-3D, demonstrates how the data-to-image approach for analysis of biological data can effectively incorporate different types of omics data and preserve the explicit connections between these layers by placing them in the same positions but in different channels of an input image. As was demonstrated in our previous work, once converted to image form data becomes suitable for use with image-specific convolutional neural network architectures. This study is the first to use this type of omics integration and likewise the first to apply this type of approach to the problem of personalized cancer drug response prediction. Our results have shown that DeepInsight-3D can outperform previously proposed methods and can also be very powerful way to discover underlying important genes, which can then be interpreted to understand the decisions made by the classifier and also identify key biological processes of potential interest. In future work, we would extend this approach by using a regression layer in place of a classification layer in the CNN net to enable us to utilize continuous output values. We would also look at integrating the transformer model for our future work. Presently, transformer models are predominantly used in NLP areas where input signals are time-based data. We will also look if this can be taken further to time-independent data.

## Methods

This section covers the proposed DeepInsight-3D methodology. The model consists of the following constituents (1) image transformation by DeepInsight-3D, (2) ResNet-50 model of CNN architecture, (3) class-based CAM to find activation maps, and 4) element decoder to decode genes (Fig. 1). These procedures are described hereunder.

### DeepInsight-3D: conversion of multi-layered tabular data to image for CNN

Let a multi-layered sample be depicted by ${x}_{ij}^{l}$, where $i$ represents elements or features, $j$ represents samples, and $l$ represents layers. Therefore, an $l$-th layer data can be depicted as ${M}_{l}={x}_{ij}^{l}$ for $i=\mathrm{1,2},\dots ,d$, $j=\mathrm{1,2},\dots ,n$ and $l=\mathrm{1,2},\dots ,L$, where $d$ is the dimensionality of the data, $n$ is the number of samples, and $L$ is the total number of layers. For multi-omics data in this work, $L=3$, which gives a multi-layered dataset $M=\left\{{M}_{1},{M}_{2},{M}_{3}\right\}\in {\mathbb{R}}^{d\times n\times L}$. The DeepInsight model^21^ converts non-image data ${M}_{l}$ to image data ${E}_{l}$. The size of an image sample is $p\times q$. The DeepInsight transform consists of manifold techniques such as t-SNE^31^, UMAP^72^ or Kernel PCA^73^, convex hull algorithm, rotation of Cartesian coordinates, finding pixel locations and mapping of elements to these pixel locations. In the case of t-SNE, it constructs a probability distribution over a pair of samples so that similar samples have greater probabilities and dissimilar samples have lower probabilities. Then similarly the probability distribution is found in the 2D plane. Thereafter, minimization of Kullback–Leibler divergence between the two distributions are performed.

These manifold techniques are not used in a usual manner as it is not required to visualize samples but the genes or elements. In this respect, the transpose of data ${M}_{l}$ is used to find ${P}_{l}$. Many of these techniques can project data to a 2D plane (DeepInsight-3D does not require 3D or higher dimensional projection). Therefore if $d>2$ and $n>2$ then it is possible to find 2D framework for ${M}_{l}$. We can obtain pixel locations by

1 $$P_{l} = {\mathcal{H}}\left( {M_{l} } \right)\quad {\text{for}}\quad l = 1, \ldots L$$

where $P_{l}$ is the pixel locations of layer $l$, ${\mathcal{H}}$ denotes the DeepInsight transform to find pixel locations and $M_{l}$ is an $l$-th layer of the training set (e.g. gene expression data). Note, the transpose in Eq. (1) is not explicitly shown. Once the framework of the locations is discovered using Eq. (1), elements can be mapped to find the corresponding images, such as

2 $$e_{j} = {\Phi }\left( {x_{ij}^{l} } \right)\quad {\text{for}}\quad j = 1, \ldots ,n\quad {\text{and}}\quad i = 1, \ldots ,d$$

where $\Phi$ maps a non-image sample $x\in {\mathbb{R}}^{d}$ to an image sample ${e}_{j}\in {\mathcal{F}}^{p\times q}$, here $\mathcal{F}$ is a pixel-coordinates system, and, $p$ and $q$ are sizes of rows and columns, respectively. For simplicity, the superscript $l$ is ignored on ${e}_{j}$. From Eq. (2) we get $\Phi :x\to e$. The transformation $\Phi$ also normalizes the values between $[\mathrm{0,1}]$ or $[\mathrm{0,255}]$. In this work, norm-2 has been employed which was introduced in ^21^.

Thus, the first layer of image data ($l=1$) obtained from Eq. (2) is

3 $$E_{1} = \left\{ {e_{1} ,e_{2} , \ldots ,e_{n} } \right\}$$

However, this dataset obtained from Eq. (3) is for layer $l=1$. For $l=2$, we did not compute the transform $\mathcal{H}$, however, only Eq. (2) has been used to find ${E}_{2}$. Similarly, for $l=3$, we can obtain the dataset ${E}_{3}$ from Eq. (2). Therefore, for $l=1,..,L$, we get a multi-layered image dataset with common pixel locations ${P}_{1}$. In this work, $L=3$, so we get a 3D colored image of a multi-omics sample.

In the above model, it has been assumed that information from layer 1 is more than the other two layers, and that’s why all the other samples of the remaining two layers also mapped on ${P}_{1}$. If it cannot be determined which layer has more information compared to others, then all the layers can be used simultaneously to find common pixel locations. In that case, transform $\mathcal{H}$ for $l=1,\dots ,L$ will be applied. However, it would produce multiple pixel locations (${P}_{1}\dots {P}_{L}$) and we need to find the common pixel locations from these pixel locations. This requires a two-stage process and is implemented in the DeepInsight-3D package by setting up the parameter *Parm.FeatureMap* to ‘0’. This option has not been used in this work, nonetheless, it has been detailed further in Figure S5 (Supplement File 1).

### CNN architecture for classification and feature selection

In this work, ResNet-50 has been used for CNN. For feature selection, we have incorporated class-activation maps (CAMs)^32^. However, other series nets supported by CAM can be used. ResNet-50 has a fixed input image size of $224\times 224\times 3$. However, different image sizes can be used, as package resizes and corrects the size according to the requirements of the net. The last ReLu layer has been used to find activation maps. The activation maps express the region of interest for decision making. It provides 3 colored layers in order of importance as red, yellow and blue. Since the red zone is the most informative, it has been used for feature selection purposes using the element decoder (Fig. 1). The training set and validation set are used to estimate and validate the model. The test set is used to evaluate the performance of the trained model. For CAM, only the training set has to be used to compute activations. The default values of hypermeters of CNN net, such as momentum, L2 regularization and initial learning rate have been used (as per version 2 of the DeepInsight package https://alok-ai-lab.github.io/DeepInsight/). However, the Bayesian optimization technique has been employed for Cisplatin to tune the hyperparameters. Further description is given in Supplement File 1.

### Class activation maps (CAMs) and element decoder

CAMs are computed for each image sample from the training set ${e}_{j}$. CAM produce 3 colors and if we denote ${R}_{j}$ as the computed CAM values of the red zone for a sample ${e}_{j}$, then ${R}_{j}>threshold$ depicts a region of interest for this sample. Since samples ${e}_{j}$ falls in different classes (here respondents and non-respondents), we can take an average of ${R}_{j}$ over the samples of a class. Therefore, class-based CAM can be computed as

4 $$avgR_{i} = \frac{1}{{n_{i} }}\mathop \sum \limits_{{j \in \omega_{i} }} R_{j} \quad {\text{for}}\quad i = 1, \ldots ,c$$

where ${\omega }_{i}$ denotes $i$-th class, $c$ is the number of classes (here 2), and ${n}_{i}$ is the number of training samples in this class.

For class-based CAMs, instead of taking ${R}_{j}>threshold$, one can consider $avg{R}_{i}>threshold$ from Eq. (4). Under this activated region, element decoder finds the gene subset. The decoder will locate the argument or index of a pixel falling under this region. A pixel ${p}_{k}$, located at $({a}_{k},{b}_{k})$ is defined by normalized value $[\mathrm{0,1}]$. However, depending upon the compression, it may contain one gene, more than one gene, or no gene. Searching all the pixels under the activated region (as defined by Eq. (4)), would reveal a list of selected genes. This procedure will provide class-based features (or genes or elements), however, some elements could be common across different classes.

Let ${G}_{i}$ be the gene subset found from the $i$-th class, then the overall selected genes are denoted as

5 $$G = \cup_{i = 1}^{c} G_{i}$$

### Experimental setup

We used the same setup of datasets as done in^2^, where training sets were collated from GDSC cell lines resource^3^. The test sets were collated from TCGA patients with the drug response^74^ and PDX encyclopedia resource^7^.

The data was downloaded from the Zenodo repository (https://zenodo.org/record/4036592) and correlated into the seven testing and training using R.

The training sets were from GDSC and the independent test were from PDX/TCGA. To train and validate the model, seven datasets from the GDSC collection are employed. There are 389 training samples overall for Paclitaxel, 844 for Gemcitabine, 856 for Cetuximab, 362 for Erlotinib, 829 for Docetaxel, and 829 for Cisplatin. The training set was split into two smaller training set and a validation set in a ratio of 90:10. The PDX/TCGA resource is used to obtain the independent test sets. The number of test samples for Paclitaxel (PDX) is 43, Gemcitabine (PDX) is 25, Cetuximab (PDX) is 60, Erlotinib (PDX) is 21, Docetaxel (TCGA) is 16, Cisplatin (TCGA) is 66 and Gemcitabine (TCGA) is 57. The training sets were subdivided into 90:10 ratio for training the model and validating the trained model. The separate test set was never used during the training and/or validation process. It has been used at the end to provide the evaluation parameters from the trained model. All the three layers, expression, CNA and mutation, are used to transform into colored images by the Deepinsight-3D model and ResNet-50 is used to find the classification performance.

Various parameters such as t-SNE distance, different types of CNN nets and maximum epochs were estimated using the validation set of a drug dataset (see Table S1, Supplement File 1). The hyperparameters (L2regularization, momentum and initial learning rate) were obtained from DeepInsight version 2 (where the Bayesian optimization technique had been used to tune the hyperparameters on an RNAseq dataset). Thereafter, applied to all the datasets. For Cisplatin, the results were unsatisfactory; therefore, the Bayesian optimization technique was applied with 10 maximum objectives. The hyperparameters corresponding to the best performance objective function on the validation set was used to evaluate the test set (see Table S2, Supplement File 1). The training and validation sets were artificially augmented during the training phase of CNN net (ResNet-50). For augmentation, a sample is generated by averaging any two samples having the same class label.

Test samples have two labels, non-responders (NR) and responders (R). The test set labels are exactly the same as^2^ and are shown in Table S6 (Supplement File 1). The total samples used for training models are also derived from GDSC resource, same as^2^, however, the number of NR and R may be different. For all the training sets, first we applied a median of $\mathrm{log}IC50$ to separate NR and R labels. This attempt balanced the NR and R samples in the training sets. However, in the case of Cisplatin, the validation accuracy was not promising, and so we then applied ‘mean’ to separate the labels.

Table S6 (Supplement File 1) shows that all the datasets used in this paper have gene count between 13,039 and 15,500. On the other hand, the number of training samples is between 362 and 856. This leads to the ‘curse of dimensionality’ problem or high-dimensional problem. Figure 1 provided a pipeline to solve this problem (and its related discussion) that high-dimensional tabular data is first converted to colored images by DeepInsight-3D. After that, CNN is used to get the prediction.

As it can be observed that the number of samples is very limited for all the drug response data, we augmented the training and validation sets during the training phase of CNN.

All the experiments were done on Intel Xeon Gold 5220R Server (2.2 GHz) with 24 CPU cores and 2 parallel NVIDIA A100 PCIe GPUs (CUDA cores: 6912 with 40 GB GPU memory on each A100 GPU). The operating system used was Linux (Ubuntu Desktop version 20.04).

### Pre-processing of mutation data

Cancer mutation data is most often extremely sparse, meaning that only a small number of different genes have consequential mutations in each sample. This presents a unique challenge when using it with a CNN classifier—as most inputs in this channel would be zero, it can result in inefficient use of information in that layer due to “dead” artificial neurons^75^. To counter this, we have used guilt-by-association principle to propagate the likely impact of mutations by using protein–protein interaction network. Briefly, the goal of this approach was to assign some part of an “impact” for each actual mutation to proximal genes in the network, as these are likely to be involved in similar biological functions. In this way, some meaningful value is assigned to each gene in all situations, while the information about actual mutations is still preserved by assigning to them the highest possible score. Note that the fine calibration of the impact score is not necessary for this use-case, as neural network is able to discover its own optimal weighting as long as the generated distribution is consistent across all of the training set.

This was done by mapping all of the genes in the dataset to the corresponding proteins of the protein interaction network obtained from STRING database v11.0^76^. A diffusion state distance matrix was calculated for the network based on the original definition of this distance metric^77^. Then, each node was assigned a score equal to the normalized inverse distance value of the closest mutated gene. In this way, the approach has facilitated the identification of possible functionally equivalent mutations as well as mutation hotspots, which have also been demonstrated to be an important network-based feature potentially predictive of clinical outcomes^78^.

### Model evaluation

In order to validate DeepInsight-3D, the training sets (besides the test sets) were subdivided into two sets with a 90:10 ratio. The larger set was employed to estimate the model and the smaller set was applied to validate it. The AUC was computed on the test set. In general, the default parameters of DeepInsight (version 2) were employed (https://alok-ai-lab.github.io/DeepInsight/) for this method with a few variations (see Table S1, Supplement File 1 for details). Some important parameters were norm-2 normalization (log transform) ^21^, t-SNE to obtain a 2D plane for gene expression data, and that CNA and mutations were mapped to the 2D plane obtained by gene expression, as it is generally considered that gene expression has more information compared to the other profiles. For CNN, we applied a pre-trained ResNet-50. This transfer learning helped to achieve promising results. In order to have faster training, default parameters were applied for all the datasets, and the obtained performance was satisfactory. However, for Cisplatin, we did not get promising results. Therefore, for Cisplatin, the Bayesian optimization technique of hyperparameter tuning was applied for ResNet-50. The hyperparameters that best performed over the validation set have been used for the test set (see Table S2, Supplement File 1 for details).

### Finding gene subsets through an iterative process

The number of genes in genomic or multi-omics data is typically very large, making it difficult to put all of them into a finite image size due to fixed technology limits. In this instance, quantized images are unavoidable, meaning that specific image pixels will carry several genes in a single spot. This leads to another issue of selecting a gene from those batch genes (where batch gene refers to a set of two or more genes having the same pixel location in the frame). To address this overlapping issue up to some extent, DeepInsight-3D can be run iteratively to gradually select the elements. The initial iteration will identify a subset of elements that can be utilized as input in subsequent iterations to find a smaller subset of genes or elements.

### Functional annotation and interpretation of identified gene sets

The analysis described above resulted in two gene lists (one each for responder and non-responder class) from each trained model that contained the genes identified as important for classifying training samples into a respective category. Functional interpretation of the recovered gene subsets was done individually, by mapping them onto metabolic and signaling pathways as defined by Reactome. This was followed by gene set enrichment analysis done using Fisher’s exact test with a Benjamini–Hochberg false discovery rate correction. The analysis was further extended using Ingenuity Pathway Analysis software from QIAGEN Digital Insights, which is backed by an extensive manually-curated knowledgebase. Owing to its more extensive collection of data and more detailed pathway definitions, IPA has allowed identification of a much larger set of additional significant pathways. This analysis was done both for the metabolic and phosphorylation pathways that were filtered to only include direct links with experimental evidence.

## Supplementary Information

Supplementary Information 1.

Supplementary Information 2.

Supplementary Information 3.

## Data Availability

All the datasets used in this paper can be downloaded from the Zenodo repository https://zenodo.org/record/4036592.

## Code availability

DeepInsight-3D software package (in Matlab), a dataset, installation instructions and user-manual are available from the GitHub link https://github.com/alok-ai-lab/DeepInsight3D_pkg. The example PDX_Paclitaxel dataset is also separately available from the link http://emu.src.riken.jp/DeepInsight/download_files/dataset1.mat , note the size is 88 MB. The following links for other related packages can be accessed via http://www.alok-ai-lab.com/tools.php and/or http://emu.src.riken.jp/.
